# Blocking ROR1 enhances the roles of erlotinib in lung adenocarcinoma cell lines

**DOI:** 10.3892/ol.2019.10643

**Published:** 2019-07-22

**Authors:** Hui-Li Wang, Yan-Chun Liu, Ming-Peng Long, Chuan Zheng, Jia-Hui Yang

**Affiliations:** 1 School of Basic Medicine, Chengdu University of Traditional Chinese Medicine, Chengdu, Sichuan 611137, P.R. China; 2 State Key Laboratory of Phytochemistry and Plant Resources in West China, Kunming Institute of Botany, Chinese Academy of Sciences, Kunming, Yunnan 650201, P.R. China

**Keywords:** receptor tyrosine kinase-like orphan receptor 1, lung adenocarcinoma, erlotinib resistance, AKT/mammalian target of rapamycin, small interfering RNA

## Abstract

Treatment strategies involving tyrosine kinase inhibitors (TKIs) for patients with non-small cell lung cancer (NSCLC) with epidermal growth factor receptor (EGFR) mutations have advanced significantly; however, challenges still remain regarding the development of resistance. It has been reported that receptor tyrosine kinase-like orphan receptor 1 (ROR1) acts as a hepatocyte growth factor receptor (MET) and c-Src substrate, and that the extracellular domain of ROR1 is associated with EGFR to sustain EGFR-ERBB3-PI3K signaling. Our previous study reported that blocking ROR1 significantly decreased the activity of key signal molecules in the AKT/mammalian target of rapamycin (mTOR) signaling pathway, which was associated with a significant increase of apoptosis and significant decrease of proliferation of lung adenocarcinoma cells. The present study hypothesized that inhibiting ROR1 could potentially prevent erlotinib resistance in NSCLC cell lines. Investigations were performed with two erlotinib-resistant cell lines XLA-07 and NCI-H1975, and an erlotinib-acquired-resistant cell line PC-9erlo, which was developed from its parental cell line PC-9. It was identified that the inhibition of ROR1 via small interfering RNA treatment significantly improved the anti-proliferation and apoptosis-inducing roles of erlotinib in TKI-resistant tumor cells. This was in accordance with the activity of key molecules of the AKT/mTOR signaling pathway, including glycogen synthase kinase-3α/β (GSK-3α/β), phosphatase and tensin homolog (PTEN), AKT, mTOR and ribosomal protein S6 kinase β-1 (p70S6K). The current data suggest that targeting ROR1 is a potential novel treatment strategy for patients with ROR1-positive NSCLC, particularly those with acquired resistance to EGFR-TKI.

## Introduction

Cancer is a significant global health problem; in 2017 it was predicted that 600,920 cancer-associated mortalities would occur in the USA and 26% of those cases would be associated with lung cancer (1). Non-small cell lung cancer (NSCLC) accounts for >80% of primary lung cancer cases (2). The identification of specific molecular targets against NSCLC has promoted a shift towards personalized treatment strategies in clinics (3,4).

Abnormal activation of the epidermal growth factor receptor (EGFR) signaling pathway has been reported in NSCLC, which leads to the activation of subsequent intracellular signaling pathways, including the phosphoinositide 3-kinase (PI3K)/AKT and mitogen-activated protein kinase 1 (MAPK) signaling pathways, which serve important roles in the proliferation, differentiation, migration and apoptosis of tumor cells (5,6). To attenuate the effects of EGFR-mediated proliferation of cancer cells, EGFR tyrosine kinase inhibitors (EGFR-TKIs) that specifically bind to the tyrosine kinase domain of EGFR and inhibit its activity have been widely administered clinically (7).

Erlotinib is a first-generation EGFR-TKI for patients with EGFR mutation-positive lung adenocarcinoma. Erlotinib elicits effective treatment responses, however, these responses are lost after a long period of time due to acquired resistance (7,8). The most common mechanism of acquired resistance is a secondary T790 mutation in EGFR termed EGFR T790M. Other mechanisms include stimulation of alternative pathways either by activation of other kinases, including hepatocyte growth factor receptor (MET) and human epidermal growth factor receptor 2, or alterations of key components in the EGFR pathway, including activation of phosphatidylinositol-4,5-bisphosphate 3-kinase or loss of phosphatase and tensin homolog (PTEN), which eliminate the requirement for EGFR-mediated tumor cell activation (9–14). To overcome EGFR-TKI resistance in lung cancer, numerous combinatorial strategies have been reported that demonstrate effective results and provide promising strategies to prevent resistance and potentially reduce the toxicity of both agents (15–24).

The receptor tyrosine kinase-like orphan receptor 1 (ROR1) is a type 1 transmembrane protein expressed on the plasma membrane (25). Previous studies have demonstrated that ROR1 is an oncogene that is highly expressed in numerous types of hematologic malignancy and several types of solid tumor, including lung cancer (26,27). ROR1 acts as a partner for the oncogenic tyrosine kinase MET and sustains the MET-driven transformed phenotype (28). ROR1 is also required to sustain the association between EGFR and receptor tyrosine-protein kinase erbB-3 (ERBB3), the activation of ERBB3, consequentially, making ROR1 an ideal target for therapies against EGFR-TKI resistance in lung adenocarcinoma (29).

Our previous investigation of patients with lung adenocarcinoma revealed that >60% of tumor tissues expressed ROR1, and inhibition of ROR1 significantly downregulated the proliferation of NSCLC cells and induced cell apoptosis (27). The current study analyzed the effect of ROR1 inhibition combined with erlotinib on the induction of apoptosis and the inhibition of proliferation via the AKT/mTOR signaling pathway. In summary, the present study provided a novel therapeutic strategy to increase the sensitivity of ROR1^+^ lung adenocarcinoma to erlotinib treatment.

## Materials and methods

### 

#### Cell lines and cell culture

The NSCLC cell line NCI-H1975 was kindly provided by the Stem Cell Bank, Chinese Academy of Sciences. The human lung cancer cell line XLA-07 was a gift from Professor Yong Duan (First Affiliated Hospital of Kunming Medical University, Kunming, China) (30) and the PC-9 cell line was a gift from Dr Jun Zhang (Shanghai Pulmonary Hospital, Shanghai, China) (31). The cells were cultured at 37°C in a 5% CO_2_ incubator (Panasonic Healthcare,) in RPMI-1640 (HyClone; GE Healthcare Life Sciences) supplemented with 10% fetal bovine serum (Beijing Transgen Biotech Co., Ltd.) and 1% penicillin/streptomycin (HyClone; GE Healthcare Life Sciences).

#### Establishment of an acquired erlotinib-resistant cell line termed PC-9erlo

The acquired erlotinib-resistant cell line termed PC-9erlo was established from the parental cell line PC-9. Briefly, 2×10^6^ cells were seeded in a 10 cm^2^ dish and then exposed to 10 µM erlotinib (Cayman Chemical Company). Following incubation at 37°C for 48 h the cells were washed with 1X PBS and then cultured in complete medium without erlotinib. To acquire and maintain the erlotinib resistance of PC-9erlo cells, the cultured cells were collected and gradually exposed to increasing concentrations of erlotinib (0.1 µM for 2 months, 0.5 µM for 2 months, 1.25 µM for 2 months and 2.5 µM for 2 months). Following the 2 months of exposure to 2.5 µM erlotinib, the half-maximal inhibitory concentration (IC50) value of erlotinib in PC-9erlo was 2.62±0.82 µM.

#### Silencing of human ROR1

ROR1 small interfering RNA (siRNA), termed siROR1, was obtained from Ambion (Thermo Fisher Scientific, Inc.). The sequence of siROR1 was sense 5′-GUACUGCGAUGAAACUUCATT-3′. The method of silencing ROR1 with siRNA was as previously described (27) with modifications. Briefly, the cells were seeded in 96- or 6-well plates, and incubated in a CO_2_ incubator for 12 or 20 h. The cells were then transfected with siROR1 or a non-targeting control siRNA (siNC; Invitrogen; Thermo Fisher Scientific, Inc.) at a concentration of 20 or 25 nM, followed by culture in serum-free medium for 6 h. Transfections were performed in Opti-MEM reduced serum medium (Thermo Fisher Scientific, Inc.) using Lipofectamine RNA iMAX (Thermo Fisher Scientific, Inc.) according to the manufacturer's protocol.

#### ROR1 expression analysis

Expression of ROR1 in different cell lines was examined by flow cytometry. Briefly, the cells were collected 72 h after ROR1 silencing with 25 nM siROR1 (cat. no. 4457298; Ambion; Thermo Fisher Scientific, Inc.) using Lipofectamine RNAiMAX (Thermo Fisher Scientific, Inc.) for 6 h at 37°C and washed twice with ice-cold PBS. R12 is a chimeric rabbit/human anti-ROR1 monoclonal antibody with a hemagglutinin (HA) tag that was developed in Christoph Rader's laboratory by the corresponding author (Jia-Hui Yang, School of Basic Medicine, Chengdu University of TCM, Chengdu, China) (32). R12 (5 µg/ml) or normal human IgG antibodies (5 µg/ml; cat. no. 009-000-003; Jackson ImmunoResearch Laboratories, Inc.) were added to the cells and incubated at 4°C for 30 min. Following washing, 5 µl PE-conjugated anti-HA monoclonal antibody (cat. no. 130-098-806; Miltenyi Biotec, Inc.) was added and incubated at 4°C for 30 min. Finally, the cells were suspended in 500 µl flow cytometry buffer and analyzed using an Accuri C6 flow cytometer (BD Biosciences). Data were analyzed using FlowJo v7.6.2 software (FlowJo LLC). The inhibition rate of ROR1 expression level in different cell lines was calculated using the formula: [value (siNC)-value (background)]-[value (siROR1)-value (background)]/[value (siNC)-value (background)] ×100% (value, value of mean fluorescence intensity of cells in different treated groups).

#### MTS cytotoxicity assay

Cells were seeded in 96-well plates at 4–6×10^3^ per well and transfected with 20 nM siROR1 or siNC, then cultured for 48 h prior to treatment with a range of concentrations (0, 1.25, 2.5, 5, 10, 20 µM) of erlotinib (Cayman Chemical Company). Cell cytotoxicity was examined using the CellTiter 96^®^ AQueous one solution reagent (Promega Corporation) with the following steps: 20 µl of the reagent was added to each well and cells were incubated in the dark at 37°C for 1 h. Cell viability was examined by measuring absorbance at 490 nm using a microplate reader. Experiments were performed in triplicate. Cell growth ratio values were calculated using the formula: 100× [A490 (sample, T)-A490 (sample, T0)]/[A490 (control, T)-A490 (control, T0)] (T, value of absorbance at 490 nm of wells with different treatment cells; T0, value of absorbance at 490 nm of wells without cells). Cell images in different treated groups were observed by inverted optical microscope (magnification ×100; XD-30; Sunny Optical Technology Co., Ltd.).

#### Apoptosis assay

Cell apoptosis was analyzed using flow cytometry. Cells were seeded in 6-well plates at 1.2–1.8×10^5^ cells/well and appropriate concentrations of erlotinib (NCI-H1975, 2.5 µM; PC-9erlo, 2.5 µM and XLA-07, 10 µM) were added to the wells 48 h after 20 nM siRNA transfection. The plates were then incubated at 37°C for 2–5 days. Cells were collected and washed, then incubated with 5 µl FITC-conjugated Annexin V and propidium iodide (BD Biosciences) in the dark for 15 min at room temperature. Cell apoptosis was measured using a FACSCalibur flow cytometer (FACSCalibur; BD Biosciences).

#### Bio-Plex pro assays

Multiple proteins and the AKT signaling pathway were evaluated using a Bio-Plex signaling AKT 8-plex panel (cat. no. LQ00006JK0K0RR) and a Bio-Plex pro signaling reagent kit (cat. no. 171304006M) both from Bio-Rad Laboratories, Inc., according to the manufacturer's protocol. Briefly, 24 h after transfection with 25 nM siRNA, NCI-H1975 cells were treated with 2.5 µM erlotinib at 37°C for 24 h. The cells were lysed 48 h later with RIPA buffer (Beyotime Institute of Biotechnology) supplemented with 10% phosphatase inhibitor (Roche Diagnostics) and 1% protease inhibitor (EMD Millipore) at 4°C. Enhanced BCA Protein Assay kit (Beyotime Institute of Biotechnology) was used to analyze the protein concentration of each cell lysate. Suspended beads were added to a 96-well flat bottom plate at 50 µl per well and the plate was washed with washing buffer using a Bio-Plex Pro II Wash station (Bio-Rad Laboratories, Inc.). Subsequently, 10 µg cell lysate was added to each well. Following incubation of the plate overnight at room temperature with shaking (450 RPM), biotin conjugated detecting antibody cocktail from the kit (Bio-Plex signaling AKT 8-plex panel, cat. no. LQ00006JK0K0RR; Bio-Plex pro signaling reagent kit, cat. no. 171304006M; dilution 1:20; Bio-Rad Laboratories, Inc.) and reagent Streptavidin-PE (Bio-Rad Laboratories, Inc.) were added and measurements were obtained with the Bio-Plex 200 system (Bio-Rad Laboratories, Inc.) at a wavelength of 575 nm. Results were recorded as relative fluorescence units and data were analyzed using GraphPad Prism version 6 (GraphPad Software Inc.).

#### Western blot analysis

Cell treatment, protein extraction and quantification were performed as aforementioned. Normalized amounts of protein (approximately 30 µg each lane) were added to 12% SDS-PAGE gels, then electrically separated and transferred onto PVDF membranes (EMD Millipore). Membranes were blocked with 5% skimmed milk for 1 h at 37°C and stained with rabbit anti-phospho(p)-p70S6K (cat. no. 9205S; dilution 1:400), mouse anti-AKT (cat. no. 2920S; dilution 1:500), mouse anti-p-AKT (cat. no. 4051S; dilution 1:400) (all from Cell Signaling Technology, Inc.), mouse anti-p70S6K (cat. no. 611260; dilution 1:800) and mouse anti-Bcl-2 (cat. no. 51-6511GR; dilution 1:500) (all from BD Biosciences). Mouse anti-β-actin antibody (cat. no. HC201; dilution 1:1000; Beijing Transgen Biotech Co., Ltd.) was used as the loading control. Horseradish peroxidase-conjugated anti-rabbit antibody (cat. no. HS101-01) and anti-mouse antibody (cat. no. HS201-01) (both dilution 1:5,000; Beijing Transgen Biotech Co., Ltd.) were used as secondary antibodies. Specific proteins were detected using an enhanced Pierce ECL western blotting substrate (Thermo Fisher Scientific, Inc.). Grayscale values were measured using ImageJ v1.51s software (National Institutes of Health).

#### Statistical analysis

Data are presented as the mean ± standard error of the mean of at least three independent experiments. Statistical significance of data was determined by analysis of variance with LSD post hoc test, using GraphPad Prism version 6 (GraphPad Software Inc.). P<0.05 was considered to indicate a statistically significant difference.

## Results

### 

#### Silencing ROR1 with siRNA enhances the cytotoxicity of erlotinib in erlotinib-resistant cell lines

**Figure 1.**
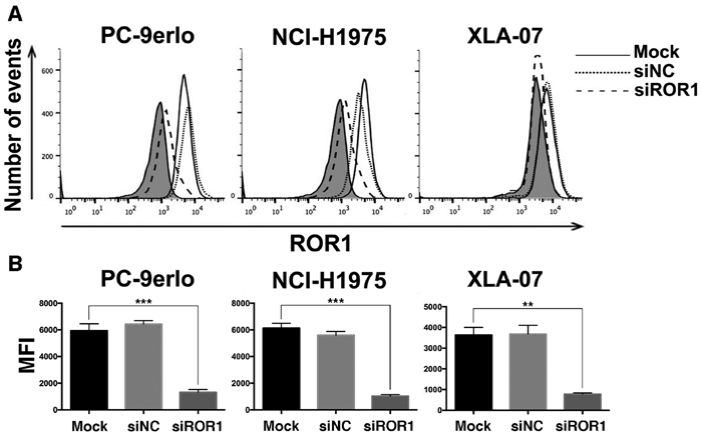
Silencing ROR1 with siRNA significantly reduces the expression of ROR1 in non-small cell lung cancer cell lines. (A) NCI-H1975, PC-9erlo and XLA-07 cell lines were treated with Mock, 25 nM siROR1 or siNC for 72 h, and ROR1 expression levels were examined using flow cytometry with R12, a chimeric rabbit/human anti-ROR1 monoclonal antibody, or normal human IgG. The background signal stained with human IgG is presented in gray. (B) MFI value of ROR1 expression in NCI-H1975, PC-9erlo and XLA-07 cell lines (**P<0.01, ***P<0.001, mock vs. siROR1). Experiments were performed 3 times (n=3). The y-axis represents the number of cells acquired by flow cytometry. ROR1, receptor tyrosine kinase-like orphan receptor 1; si, small interfering; NC, negative control; Mock, complete medium; MFI, Mean fluorescence intensity.

**Figure 2.**
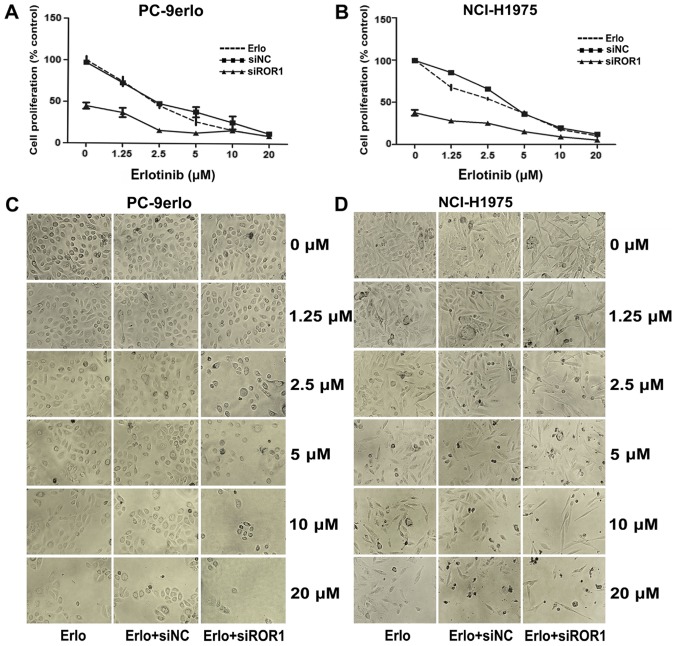
Silencing ROR1 together with erlotinib reduces cell proliferation of non-small cell lung cancer cell lines. (A) PC-9erlo and (B) NCI-H1975 cell lines were treated with erlotinib alone, erlotinib +20 nM siNC or erlotinib + 20 nM siROR1, and analyzed for growth inhibition using the MTS assay. Experiments were performed three times (n=3). Microscope images of (C) PC-9erlo and (D) NCI-H1975 cells in different treatment groups (magnification, ×100). ROR1, receptor tyrosine kinase-like orphan receptor 1; si, small interfering; NC, negative control; erlo, erlotinib.

The present study first examined ROR1 expression levels by flow cytometry following ROR1 silencing with siRNA in NCI-H1975, PC-9erlo and XLA-07 cell lines. The results indicated that the ROR1 expression level was reduced by siROR1 with all inhibition rates >75% (Fig. 1). The growth inhibitory efficacy of erlotinib following ROR1 silencing was evaluated using the MTS assay. Compared with erlotinib alone, the specific cell proliferation rates of blocking ROR1 together with erlotinib at concentrations of 1.25, 2.5, and 5 µM in PC-9erlo cells were 36.96 vs. 74.31%, 15.67 vs. 45.27%, and 12.67 vs. 26.13%, respectively. In addition, compared with erlotinib alone the cell proliferation rates of blocking ROR1 together with erlotinib at the aforementioned concentrations in NCI-H1975 cells were, 27.98 vs. 67.51%, 25.18 vs. 54.01% and 15.17 vs. 36.72%, respectively (Fig. 2A and B).

#### Blocking ROR1 enhances the apoptosis-inducing role of erlotinib in erlotinib-resistant cell lines

**Figure 3.**
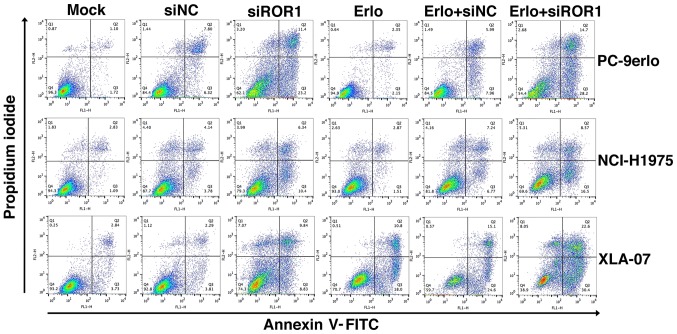
Blocking ROR1 with siRNA enhances the apoptosis-inducing role of erlotinib in non-small cell lung cancer cell lines. NCI-H1975, PC-9erlo and XLA-07 cell lines were treated with Mock, 20 nM siNC, 20 nM siROR1, erlotinib alone, erlotinib + 20 nM siNC or erlotinib + 20 nM siROR1. The concentration of erlotinib was 2.5 µM for PC-9erlo and NCI-H1975 cells, and 10 µM for XLA-07 cells. Following treatment, apoptosis of the cells was analyzed by Annexin V/propidium iodide staining. Experiments were performed 3 times (n=3). ROR1, receptor tyrosine kinase-like orphan receptor 1; si, small interfering; NC, negative control; Mock, complete medium.

**Figure 4.**
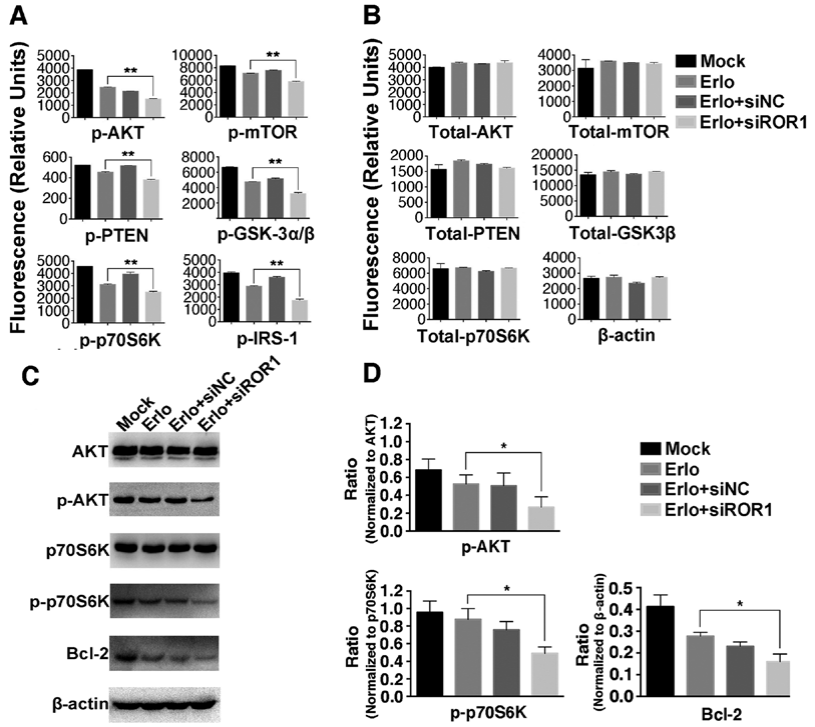
Inhibition of ROR1 has an additive role with erlotinib in the NCI-H1975 cell line via the AKT/mTOR signaling pathway. NCI-H1975 cells were treated with Mock, 2.5 µM erlotinib alone, 2.5 µM erlotinib + 25 nM siNC or 2.5 µM erlotinib + 25 nM siROR1. The (A) phosphorylated and (B) total protein levels were analyzed using the Bio-Plex signaling AKT 8-plex panel and Bio-Plex pro signaling reagent kit, because the kit did not contain antibody detecting total protein of IRS-1, β-actin was used as control instead. Values are presented as relative fluorescence units. Data are presented as the mean of three independent experiments. (C) The phosphorylated and total protein expression levels of AKT, p70S6K, Bcl-2 and β-actin were determined using western blot analysis. (D) The integrated density analysis demonstrated the changes in the expression levels of p-AKT, p-p70S6K and Bcl-2, and difference in phosphorylated protein levels was analyzed using ratios between the phosphorylated/total protein. Experiments were performed 3 times (n=3). Statistical analysis was performed using analysis of variance. *P<0.05 and **P<0.01, erlotinib treated group vs. erlotinib+siROR1 treated group. ROR1, receptor tyrosine kinase-like orphan receptor 1; si, small interfering; NC, negative control; Mock, complete medium; mTOR, mammalian target of rapamycin; IRS-1, insulin receptor substrate 1; GSK-3α/β, glycogen synthase kinase-3α/β; p, phosphorylated.

To gain further insight into the additive roles of blocking ROR1 combined with erlotinib in erlotinib-resistant cells, NCI-H1975, PC-9erlo and XLA-07 cell lines were treated with complete medium (mock group), siNC alone, siROR1 alone, erlotinib alone (Erlo), siNC plus erlotinib (Erlo+siNC) and siROR1 plus erlotinib (Erlo+siROR1). The apoptosis rates of the cells were then analyzed using flow cytometry (Fig. 3). All three erlotinib-resistant cell lines demonstrated a limited response to erlotinib alone; however significantly different apoptosis rates were revealed when treated with Erlo+siROR1 compared with that in the Erlo group (NCI-H1975, 16.5 vs. 1.51%; PC-9erlo, 28.2 vs. 2.15%; XLA-07, 30.4 vs. 18.0%). The expression level of Bcl-2 was then further analyzed as Bcl-2 is considered an important antiapoptotic protein. Using western blot analysis it was revealed that the expression level of Bcl-2 was markedly decreased in the Erlo+siROR1 group compared with that in the Erlo group (Fig. 4C and D), which indicated that the activity of Bcl-2 was downregulated.

#### Silencing ROR1 with siRNA prevents erlotinib resistance via the AKT/mTOR signaling pathway in NCI-H1975 cells

To investigate the molecular mechanisms of ROR1-silencing-enhanced cytotoxicity and the apoptosis-inducing roles of erlotinib, key molecules in the AKT/mTOR signaling pathway were analyzed in the erlotinib-resistant NCI-H1975 cell line using the Bio-Plex Pro assay (Fig. 4A and B), and since the kit did not contain an antibody detecting total protein of IRS-1, β-actin was used as control instead. It was identified that the phosphorylation levels of IRS-1, GSK-3α/β, AKT, p70S6K, PTEN and mTOR were significantly lower in the Erlo+siROR1-treated group compared with that in the erlotinib-treated group. To confirm these findings, the phosphorylation of AKT and p70S6K was further analyzed using the western blot assay (Fig. 4C and D). This revealed that the activity of AKT and p70S6K was significantly downregulated in the Erlo+siROR1-treated group compared with the Erlo treatment group alone, which was consistent with the data from the Bio-Plex assay.

## Discussion

Treatment with TKIs provides significant benefits for patients with EGFR mutations, particularly for those with lung cancer. However, the majority of patients with NSCLC will acquire resistance to first-generation EGFR-TKIs, including gefitinib and erlotinib, following 9–14 months of treatment (7). There are two central mechanisms that are involved in this process: EGFR secondary mutations and alternative signaling activation (5,6). In addition, in an EGFR-independent manner, dysregulation of other receptor tyrosine kinases (RTKs) or abnormal activation of downstream compounds have compensatory functions against the inhibition of EGFR by altering the PI3K/AKT and MAPK signaling axis. Certain studies have revealed that the proline-rich region of the intracellular domain of ROR1 is directly activated by MET and the pseudokinase domain is phosphorylated by Src (26,28). Yamaguchi *et al* (29) also demonstrated that a cysteine-rich domain of the extracellular domain of ROR1 is associated with EGFR and sustains EGFR-ERBB3-PI3K signaling. It may be beneficial to clarify whether ROR1 silencing has an additive role with erlotinib in lung adenocarcinoma, which could provide a potential new therapeutic strategy for patients with lung cancer, and TKI insensitivity and resistance.

The present study selected an erlotinib-resistant cell line NCI-H1975, which is known to be a T790M-mutant, and another erlotinib-resistant cell line XLA-07, and an erlotinib-acquired resistant cell line PC-9erlo, which was developed from its parental cell line PC-9 and mimics the situation that occurs in clinical treatment. The current results demonstrated that ROR1 inhibition plus erlotinib have additional cytotoxic effect in ROR1 positive lung adenocarcinoma cell lines. In addition, it was identified that the expression level of Bcl-2, a key regulator of antiapoptotic signaling (33), was significantly lower in Erlo+siROR1-treated cells (Fig. 4D), which was in accordance with the apoptosis-inducing role of ROR1 inhibition combined with erlotinib.

**Figure 5.**
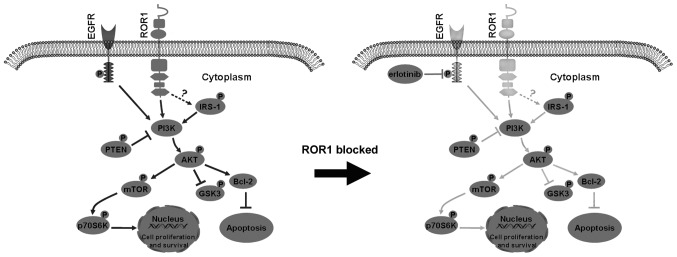
Proposed model of the combined effect of ROR1 inhibition and erlotinib treatment in non-small cell lung cancer cell lines via inhibition of the AKT/mTOR signaling pathway. Inhibition of ROR1 significantly decreased the activity of IRS-1, AKT, mTOR and p70S6K, and activated GSK-3α/β and PTEN, which are two negative regulators of PI3K/AKT signaling. This enhances cell apoptosis, and reduces cell proliferation and survival. ROR1, receptor tyrosine kinase-like orphan receptor 1; IRS-1, insulin receptor substrate 1; GSK-3α/β, glycogen synthase kinase-3α/β; p, phosphorylated; EGFR, epidermal growth factor receptor.

ROR1-mediated signaling pathways in lung cancer are not fully understood. Our previous data suggested that the AKT/mTOR signaling pathway is necessary for ROR1-mediated proliferation and antiapoptosis in lung adenocarcinoma. The AKT/mTOR signaling pathway is important for regulating cell proliferation, cancer growth and longevity (6,34,35). The present study investigated the association of the AKT/mTOR signaling pathway with ROR1 silencing against erlotinib resistance in lung cancer. Compared with erlotinib alone, phosphorylation of key molecules in the AKT/mTOR signaling pathway, including insulin receptor substrate 1 (IRS-1), glycogen synthase kinase-3α/β (GSK-3α/β), PTEN, AKT, mTOR and p70S6K, was significantly lower when ROR1 was silenced in combination with erlotinib. This supports the hypothesis that inhibiting the ROR1-mediated signaling pathway could partially overcome erlotinib-resistance via upregulation of the activity of IRS-1, AKT, mTOR and p70S6K, and downregulation of the activity of GSK-3α/β and PTEN, which are negative regulators of PI3K/AKT (Fig. 5). Inhibiting ROR1 with small molecules and monoclonal antibodies, or inhibiting the key regulators involved in AKT/mTOR signaling could effectively increase the sensitivity of tumor cells to erlotinib.

The present study revealed that the protein expression level of IRS-1, which is involved in cell proliferation, was also significantly reduced through the inhibition of ROR1 in combination with erlotinib. The underlying association between ROR1 and IRS-1 is unclear; however, targeting IRS-1 in NSCLC has been reported to exhibit an antitumor effect in a number of studies (36–38). It remains to be determined whether interactions between ROR1 and IRS-1 directly activate IRS-1 following binding to its ligand, or whether an indirect stabilization occurs through the association of IRS-1 with insulin-like growth factor-1 receptors, thus transmitting signals to the intracellular AKT/mTOR pathway.

In conclusion, the present study identified that ROR1 is a potential target for preventing erlotinib resistance in lung adenocarcinomas via the AKT/mTOR signaling pathway. Targeting ROR1 with small molecules or immunological procedures may increase the sensitizing of tumor cells, particularly erlotinib-resistant cells, to erlotinib. Further studies should investigate the functions of ROR1 in lung adenocarcinoma to promote the development of ROR1-targeting therapies in the future.

## Data Availability

The datasets used and analyzed during the current study are available from the corresponding author on reasonable request.

## Abbreviations

ROR1: receptor tyrosine kinase-like orphan receptor 1
EGFR: epidermal growth factor receptor
TKI: tyrosine kinase inhibitor
PI3K: phosphoinositide 3-kinase
mTOR: mammalian target of rapamycin
IRS-1: insulin receptor substrate 1
GSK-3: glycogen synthase kinase 3
ERBB3: receptor tyrosine-protein kinase erbB-3
MAPK: mitogen-activated protein kinase 1
p70S6K: ribosomal protein S6 kinase β-1
PTEN: phosphatase and tensin homolog
MET: hepatocyte growth factor receptor
